# Expression of the *high-affinity K^+^ transporter 1* (*PpHKT1*) gene from almond rootstock ‘Nemaguard’ improved salt tolerance of transgenic Arabidopsis

**DOI:** 10.1371/journal.pone.0214473

**Published:** 2019-03-26

**Authors:** Amita Kaundal, Devinder Sandhu, Marco Duenas, Jorge F. S. Ferreira

**Affiliations:** 1 USDA-ARS, US Salinity Lab, Riverside, California, United States of America; 2 College of Agriculture and Applied Sciences, Utah State University, Logan, Utah, United States of America; 3 College of Natural and Agricultural Sciences, University of California Riverside, Riverside, California, United States of America; National Taiwan University, TAIWAN

## Abstract

Soil salinity affects plant growth and development, which directly impact yield. Plants deploy many mechanisms to cope with, or mitigate, salt stress. One of such mechanism is to control movement of ions from root to shoot by regulating the loading of Na^+^ in the transpiration stream. The high-affinity K^+^ transporter 1 (HKT1) is known to play a role in the removal of Na^+^ from the xylem and bring it back to the root. As almond is a salt-sensitive crop, the rootstock plays an important role in successful almond cultivation in salt-affected regions. We currently lack knowledge on the molecular mechanisms involved in salt tolerance of almond rootstocks. In this study, we complemented the Arabidopsis *athkt1* knockout mutant with *HKT1* ortholog (*PpHKT1)* from the almond rootstock ‘Nemaguard’. Arabidopsis transgenic lines that were generated in *athkt1* background with the constitutive promoter (PpHKT1OE2.2) and the native promoter (PpHKT1NP6) were subjected to different salt treatments. Both transgenic lines survived salt concentrations up to 120 mM NaCl, however, the mutant *athkt1* died after 18 days under 120 mM NaCl. At 90 mM NaCl, the dry weight of *athkt1* decreased significantly compared to the transgenic lines. Both transgenic lines showed significantly longer lateral roots compared to the *athkt1* mutant at 80 mM NaCl treatment. The transgenic lines, PpHKT1OE2.2 and PpHKTNP6 had lower electrolyte leakage and higher relative water content compared to *athkt1*, suggesting that transgenic plants coped well with increased salt concentration by maintaining the integrity of the membranes. The expression analyses showed that *PpHKT1* was induced in PpHKT1OE2.2 and PpHKTNP6 lines under salt treatment, which confirmed that both over-expression and native expression of *PpHKT1* in the Arabidopsis mutant can complement salt tolerance function.

## Introduction

Global climate change has led to longer, more frequent and severe drought periods [1]. These droughts have led to a reliance on low-quality well water and unsustainable irrigation practices for agriculture, which combined lead to increased soil salinity [2]. It has been predicted that salinization may impact 50% of the arable land by 2050 [3]. The problem of soil salinity is a scourge for the global agricultural productivity. According to recent estimates, about 23% of worldwide farmland is affected by salt and crop losses due to salinity are estimated to be in tens of billions US dollars per year [4]. The United States is one of the main countries with agricultural lands affected by salinity. In the Central Valley of California alone, crop losses were estimated to be over $ 3.7 billion in 2014 [2].

The higher salt concentration affects plants in three different ways: osmotic stress, ionic stress and nutrient deficiency [4]. Higher concentrations of salt increase the water potential of roots reducing the water uptake by plants. The ionic stress sets in later by the accumulation of Na^+^ in the plant tissues that inhibits the metabolism. While Na^+^ is also an essential macronutrient, large influxes of Na^+^ from the roots to the rest of the plant can create stress in the plant’s internal environment, often leading to disruption of ideal cellular cytoplasmic conditions that become fatal to plants [5]. High Na concentrations in plant tissue lead to K deficiency due to imbalance in Na^+^/K^+^ homeostasis [4, 6]. Na^+^ and K^+^ are similar in size and charge, which in certain cases results in K^+^ channels in cell membranes being permeable to Na^+^ as well [7]. Potassium is an important cofactor for many enzymatic reactions, such as ATP production, and an important component of the opening and closing of stomata [8].

Plants have developed various mechanisms to inhibit the uptake of ions, their transport to shoots or sequestration inside vacuoles [9]. There is a complex network of ion channels, pumps and transporters for ion influx, efflux, inhibition of ion transport to shoots by xylem loading and ion compartmentalization to protect plant tissues from their deleterious effects [4]. The excess salt in soil leads to the uptake of Na^+^ in the cytoplasm of root cells, but most of it is pumped back to the soil by plasma membrane-bound Na^+^/H^+^ antiporters and the genes involved in this mechanism are primarily of the *salt overly sensitive* (*SOS)* gene family [10]. The excess Na^+^ in the cytoplasm is moved into the vacuole through tonoplast bound Na^+^/H^+^ exchangers (NHX) that protect plants from harmful effects of Na^+^ [4, 11]. Studies showed that the overexpression of Vacuolar NHX1 and plasma membrane-bound SOS1 induce salt tolerance by controlling Na^+^ homeostasis in plant cells under salt stress [12].

The transport of Na^+^ from root to shoot is poorly understood, but studies involving the high-affinity potassium transporter (*HKT*) genes have brought about new insights into the mechanisms of response to salt stress. A single amino acid difference distinguishes HKT1 from HKT2, as HKT1 has affinity for Na^+^ only, while HKT2 provides an additional affinity for K^+^, depending on the concentrations of these respective ions in the environment [13]. In general, selective sodium affinity of the HKT1 transporters found in the plasma membrane of xylem cells prevent large concentrations of Na^+^ from building up in the shoot system, as shoots are known to tolerate lower Na^+^ concentrations than the roots [4]. Although *HKT1* is not directly involved in the transport of K^+^, the reduced presence of Na^+^ within the shoot indirectly leads to greater K^+^ affinity of various channels and co-transporters [14, 15]. When less Na^+^ is present within Arabidopsis plants, the affinity for K^+^ thereby increases [15]. HKT1’s role in salinity stress response is not limited to one species of plants with one specific protein, but rather many monocots and dicots that produce different transporters that may vary slightly in role and function. In rice, five *OsHKT1* variants have been identified with some members, such as *OsHKT1;5*, prominent in xylem parenchyma to control Na^+^ levels entering the shoots [15, 16], while *OsHKT1;4*, found more specifically in the leaf sheath is a significant component that controls Na^+^ levels within the leaf blades [15]. In bread wheat (*Triticum aestivum*), the locus *Kna1* on chromosome 4 is responsible for excluding Na^+^, which is later characterized as *TaHKT1;5-D* [14, 17]. In dicots, very few members of *HKT* transporters have been found. In Arabidopsis, *AtHKT1* has been widely studied and hypothesized to transport Na^+^ from the xylem parenchyma cells to the phloem sap and roots, a process that recirculates sodium to areas of the plant more tolerant to salinity [7]. It has been shown that Arabidopsis ecotypes with a higher expression of *HKT1* in roots had a smaller Na^+^ concentration in their shoots compared to those with lower *HKT1* expression [18]. Similarly, in a comparison of 12 genetic variants of alfalfa, the lines with the highest height and biomass under saline conditions showed overexpression of *HKT1* [19]. Although the role of *HKT1* has been examined in multiple dicots, little is known about its role in almonds (*Prunus dulcis*).

The United States is the world’s largest almond producer and California alone dominates the global market with 80% of the world’s almond production [20]. In 2014, almond exports contributed to $21.5 billion to California economy [20]. The almond orchard area doubled in the past two decades, making almonds the most extensive irrigated crop in California [21]. Although the use of degraded or alternate waters for irrigation is an economical option, salt concentration of these waters is a major concern for almond cultivation. The alternate use of ground water to irrigate plants in California has exposed almonds to salt stress. Like many other stone-fruit plants almonds are sensitive to salt stress. The development and growth of almonds is considerably reduced with irrigation water electrical conductivity (EC_iw_) above 1.5 dS m^-1^ and is reduced to 50% at 4 dS m^-1^ [22]. Some studies have been carried out on almond salt tolerance, but the knowledge on the molecular basis of salt tolerance is still lacking [23]. The use of rootstocks is a common practice for successful cultivation of most stone fruits. Rootstock plays an important role in the establishment of an orchard by enhancing nutrient uptake and imparting resistance to various abiotic and biotic stresses. Thus rootstock selection is extremely critical for almond cultivation in salt-affected areas.

In this investigation, we have isolated and cloned the *HKT1* (*PpHKT1)* gene from one of the most commonly used commercial almond rootstock, ‘Nemaguard’, and demonstrated its functional complementation in Arabidopsis *hkt1* mutant.

## Materials and methods

### Plant material and growth

**Fig 1:**
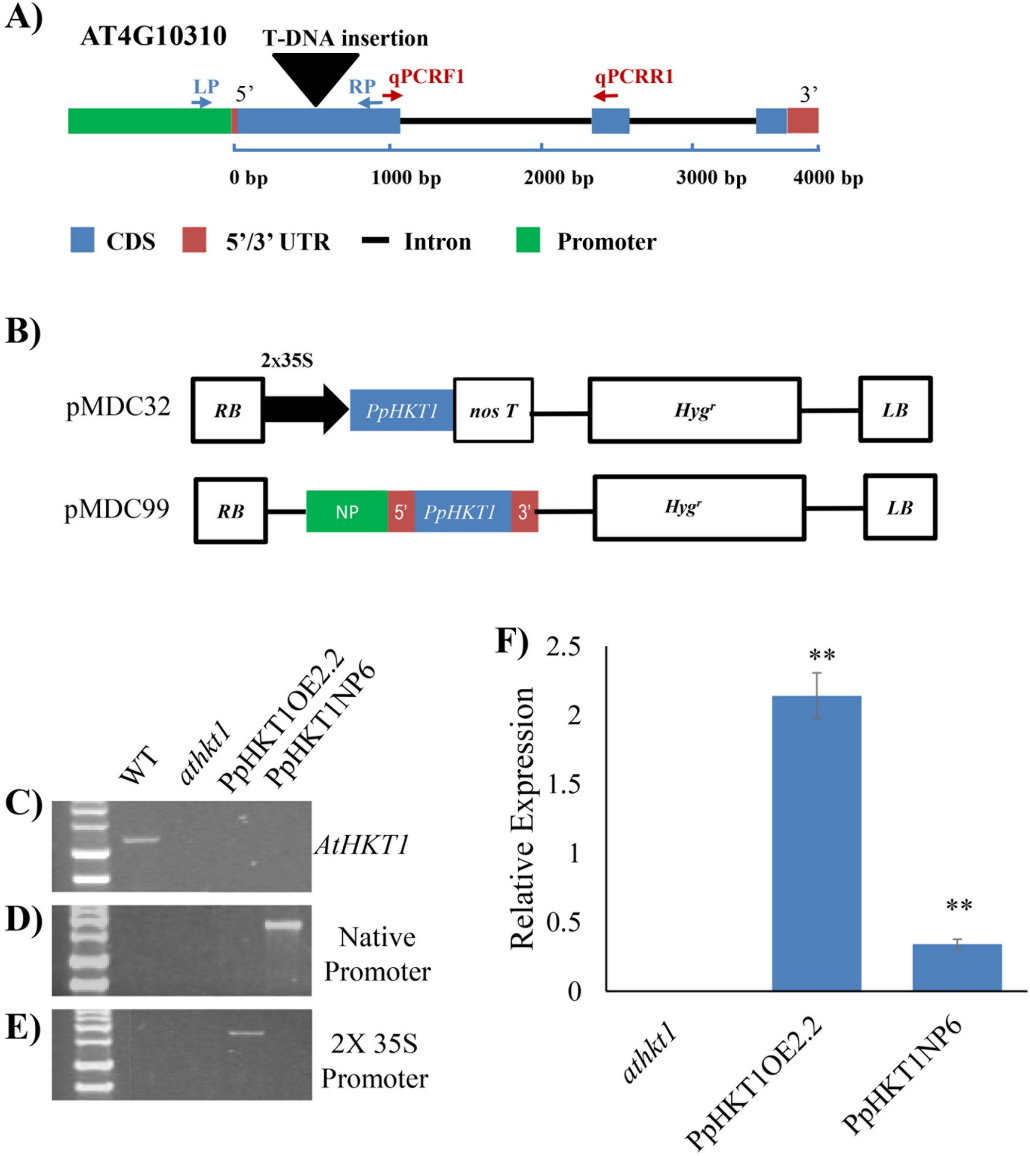
Screening of transgenic lines. **A)** Gene structure of *athkt1* with T-DNA insertion and location of primers for genotyping and qRT-PCR. **B**) Gene structure of overexpression construct in gateway vector pMDC32 and expression under native promoter in promoter-less gateway vector pMDC99. C) Screening of WT, *athkt1* mutant and the transgenic lines by amplification with GABI_795G10_LP/GABI_795G10_RP set of primers specific to the *AtHKT1* gene. WT had amplification of 1.2 Kb band and no amplification was seen in the *athkt1* mutant or the transgenic lines. **D)** Screening of Arabidopsis lines expressing *PpHKT1* under native promoter. The gDNA was amplified with PpHKT1_Start_F and PpHKT1_3’UTR_R set of primers. Only transgenic line harboring pMDC99 carrying native promoter construct amplified the 1.8 Kb amplicon. **E)** Screening of overexpressor line by amplification with 35SProm_F and PpHKT1_Stop_R set of primers. Only Arabidopsis line harboring pMDC32 carrying CDS construct amplified 1.7 Kb amplicon. **F)** Relative expression of *PpHKT1* in mutant and transgenic lines. The HKT1OE2.2 lines (overexpressor) showed ~15- fold change in expression while HKT1NP6 line (endogenous) showed ~2.5- fold change in expression with respect to the *athkt1* mutant. ANOVA single factor was used to depict the statistical differences between the *athkt1* mutant and each of the transgenic lines. P≤0.001 (**).

The *hkt1* knockout mutant Gabi Kat (GABI_795G10) was purchased from Arabidopsis Biological Resource Center (Ohio State University; www.arabidopsis.org/). The seeds were surface sterilized with 70% ethanol for 5 min, followed by 30% bleach + 0.25% Triton 100 for 5 min. Sterilized seeds were spread on ½-strength Murashige and Skoog medium containing 1.5% sucrose and 6% agar and were vernalized for 4 days at 4 °C. The seeds were then moved to a growth chamber (Conviron, Model CMP 4030, Winnipeg, Manitoba, Canada) set at 21 °C / 18 °C day/night temperatures. The day/night cycles of photoperiod set were 16 h / 8 h. The light intensity was 200 μmol m^−2^ s^−1^ and the relative humidity (RH) was approximately 50%. After one week of germination, seedlings were transferred to metro mix (SUNGRO, Horticulture Distribution, Inc., Bellevue, WA, USA) and leaf samples were collected after two weeks. The knockout mutant was screened for homozygous lines using GABI_795G10_LP/GABI_795G10_RP and GABI_795G10_LP /Lb1.3 primers (Table 1). The gene structure of T-DNA insertions and location of primers are shown in Fig 1A. Primers were designed using SIGNAL iSect Tools (http://signal.salk.edu/isects.html). The gene expressions of homozygous mutant lines were checked by quantitative Reverse Transcription—Polymerase Chain Reaction (qRT-PCR) analyses.

**Table 1:** The list of primers used in the study.

Primer	Sequence (5' to 3')
GABI_795G10_LP	AAGAAAGAGAACTTTTCCACGTG
GABI_795G10_RP	AAAGGCGCAGAAGAAAAGAAG
LBb1.3	ATTTTGCCGATTTCGGAAC
AtHKT1_qPCRF_1	AAGTGGTGAATTCGCGACA
AtHKT1_qPCRR_1	GTGTATGGAGGAAGATACATCATAAGA
At Actin_qPCR_F	GGCGATGAAGCTCAATCCAAACG
At Actin_ qPCR_R	GGTCACGACCAGCAAGATCAAGACG
AtUBQ5qPCRF	CTTACACCAAGCCGAAGAAGA
AtUBQ5qPCRR	CTCCTTCCTCAAACGCTGAA
PpHKT1_CDS_attB1	GGGGACAAGTTTGTACAAAAAAGCAGGCTctATGAAGAACTTTGCTGTTCTTGG
PpHKT1_CDS_attB2	GGGGACCACTTTGTACAAGAAAGCTGGGTcTCAAGATAGCTTCCAAGCTTTG
PpHKT1_Promo_attB1	GGGGACAAGTTTGTACAAAAAAGCAGGCTctTTGACGGTTTCTGCAATCTC
PpHKT1_Promo_attB2	GGGGACCACTTTGTACAAGAAAGCTGGGTcCAATCAGCCCAATTAGTTTAATACC
35SPromoter_F	CTATCCTTCGCAAGACCCTTC
PpHKT1_MidR	TCAGCATTAGAGTCTGGGAAATG
PpHKT1_MidF	CAGAATTGATGTAGTCAATACCAAA
PpHKT1_cDNA_R	CAAATTTCTGATAGGCAGTCAGAC
PpHKT1.3'UTR_R	CAATCAGCCCAATTAGTTTAATACC
PpHKT1_Start_F	ATGAAGAACTTTGCTGTTCTTGG
PpHKT1_STOP_R	TCAAGATAGCTTCCAAGCTTTG
PpHKT1_1950_F	TGTTGAAGAATTATAAAGACATGGG
attB1	GGGGACAAGTTTGTACAAAAAAGCAGGCTct
attB2	GGGGACCACTTTGTACAAGAAAGCTGGGTc
PpHKT1qPCR-F2	TTCAGACCATGAATTCGAGACAT
PpHKT1qPCR-R2	TATATGGTGGAAGATACATCATCACC

### Cloning of *PpHKT1*

The almond rootstock ‘Nemaguard’ (*Prunus persica*) was used for cloning the *HKT1* gene. The nucleotide sequence of the *HKT1* gene (*Prupe*.*1G067100*) was used to amplify coding sequence (CDS), cDNA and promoter region from root tissues. The CDS and 3’UTR were amplified using primers PpHKT1_Start_F and PpHKT1_3’UTR_R from the cDNA (Table 1). Both native promoter and the 5’UTR were amplified from the genomic DNA extracted from roots using primers PpHKT1_PromB1 and PpHKT1_cDNA_R (Table 1). RNA was extracted using Spectrum Plant Total RNA Kit (Sigma Aldrich, Carlsbad, CA, USA) and cDNA was synthesized using oligo dt_18_ with PrimScript 1^st^ strand cDNA synthesis kit (Takara, Bio USA, Mountain View, USA). Reverse transcription was carried out at 65 °C for 5 minutes and 42 °C for 1 hour. The native promoter was fused to cDNA containing 3’UTR by overlapping PCR, where both the amplicon, native promoter whose 3’ end sequence overlaps with the 5’ end of cDNA containing 3’UTR. Both amplicons were mixed and used as templates. The fused fragment was amplified by using forward primer PpHKT1_promB1 specific to 5’end of native promoter amplicon and reverse primer PpHKT1_promB2 specific to 3’end of cDNA containing 3’UTR amplicon and Q5 High fidelity DNA polymerase (New England Biolabs, Ipswich, MA, USA). The obtained CDS and Native promoter + cDNA were cloned in pDONR207 (Kan) gateway entry vector (Invitrogen Corporation, Carlsbad, CA, USA). The selected clones were sequenced. The CDS from pDONR207 was transferred to the gateway destination vector pMDC32 for over expression under 2X 35S promoter. The native promoter + cDNA amplicon was transferred to gateway destination vector pMDC99 for complementation studies under native promoter. The structures of both constructs are represented in Fig 1B.

### Development of transgenic lines

The *HKT1-cds*::*pMDC32* and *HKT1-NPcDNA*::*pMDC99* were transformed to *Agrobacterium tumefaciens* strain GV3101 by electroporation. Arabidopsis transformation was carried out in the *athkt1* mutant (GABI_795G10) by floral dip in 5% sucrose and 0.25% of Silvet-77 [24]. The transgenic plants were selected on hygromycin at 25 μg/ml on ½-strength MS media. Integration of the construct was confirmed by PCR analysis of hygromycin (25mg /L)-resistant T_1_ generation plants. Hygromycin-resistant T_2_ plants from five lines per construct were used for transgenic expression analysis by qRT-PCR using *PpHKT1* specific primers PpHKT1qPCR_2F/2R (Table 1). All five lines per construct were screened for the salt-tolerance assay and then one line per construct with high transgenic expression and phenotype were taken for further analysis. T_3_ progenies were used for further experiments.

### Salt-tolerance assay of transgenic Arabidopsis lines

Wild-type (WT) Arabidopsis plants, *athkt1* mutant line and T_3_ transgenic lines PpHKT1OE2.2 and PpHKT1NP6 were surface sterilized and germinated on ½ MS medium in growth chamber at 23 °C and 50% RH with 16 h / 8 h photoperiod. Twelve plants of each line were grown in soil for three weeks and then treated with 1/8 MS media as control and 1/8 MS media supplemented with 90 mM NaCl, 120 mM NaCl or 150 mM NaCl for 18 days. The concentration of NaCl supplements were increased stepwise from 90 mM NaCl to higher concentration after each treatment on alternate days. The plants were photographed 18 days after the start of the treatment. The salt treatment with 90 mM NaCl was selected for further experiments.

### Electrolyte leakage measurement

Ion leakage was determined according to a previously described method with minor modifications [25]. Seeds for Arabidopsis WT, *athkt1* mutant, T_3_ transgenic lines PpHKT1OE2.2 and PpHKT1NP6 were germinated on ½ MS media and then one-week old seedlings were transferred to soil. Three-week-old plants were irrigated with 1/8 MS (control) or 1/8 MS with 90 mM NaCl (Saline) for two weeks on alternate days. Leaf disks of 5 mm were collected from five plants of each line and divided into four replicates (3 disks per replicate). Leaf disks were rinsed in deionized water to remove surface ions and incubated in 10 ml of deionized water overnight. The sample conductivities (C1) were measured with Model 1056 digital conductivity meter (Amber Sciences, Eugene Oregon, USA). The samples were then autoclaved and cooled down to room temperature and conductivities (C2) were measured. The relative electrolyte leakage was calculated using the formula: (C1/C2) x 100%. The whole assay was repeated three times and the data were analyzed using the ANOVA single factor.

### Lateral root growth and fresh weight determination

Lateral root growth was determined using previously established method with slight modification [26]. Arabidopsis WT plants, the *athkt1* mutant line and T_3_ transgenic lines PpHKT1OE2.2 and PpHKT1NP6 were germinated on ½ MS media. Eight six-day-old seedlings /line were then transferred to MS media or MS media containing 80 mM NaCl with 3 replications. The photographs were taken 10 days after transfer and the fresh weight of each seedling was measured. The length of lateral roots was measured using ImageJ (https://imagej.nih.gov/ij/). The experiment was repeated twice.

### Relative water content (RWC)

The RWC of the transgenic lines was determined following a method described elsewhere [25]. Leaves from 2 plants of each transgenic line with and without treatment were harvested and the fresh weight (FW) was determined. These samples were then incubated in 10 ml of deionized water for 12 hours at room temperature and the turgid weight (TW) was determined. Then all samples were dried at 65 °C for 48 h, and dry weight (DW) was determined. The relative water content was calculated using formula {(FW-DW) / (TW-DW)} x 100. The experiment was conducted in three replicates.

### quantitative Reverse Transcription- PCR (qRT-PCR)

The expression analyses of *PpHKT1* and *AtHKT1* genes were done using qRT-PCR. One-week-old Arabidopsis seedlings WT Col-0, the *athkt1* mutant and the transgenic lines expressing *PpHKT1* were treated with and without 90 mM NaCl in 1/8 MS media. Whole seedlings were collected after 48 h and RNA was extracted with TRIzol reagent (Invitrogen, Carlsbad, CA, USA). DNase treatment was done to remove DNA contamination in RNA samples by DNase I enzyme (Thermo Scientific, Waltham, MA, USA). The expression analyses were carried out in BioRad CFX96 System using iTaq Universal SYBR Green One-Step Kit (Bio-Rad Laboratories, Hercules, CA, USA). Primers specific to *PpHKT1* and *AtHKT1* were developed using Prime quest tool of IDT (https://www.idtdna.com/Primerquest/Home/Index). The reaction was carried out in 10 μl of reaction containing 50 ng of total RNA, 0.75 μM of each primer, 0.125 μl iScript Reverse Transcriptase enzyme, and 5 μl of 2 × one-step SYBR Green Reaction mix. The PCR program was as follows: 50 °C for 10 min, 95 °C for 1 min, then 40 cycles of 95 °C denaturation for 10 s, 57 °C annealing for 30 s, and 68°C extension for 30 s. The Arabidopsis actin gene was used as an internal control (Table 1). The experiment was repeated twice.

## Results

### Molecular cloning of *PpHKT1* and development of transgenic lines

To study the effect of almond *PpHKT1* to salt stress, Arabidopsis transgenic lines over expressing almond *PpHKT1* under 2x 35S promoter and endogenously expressing under its own promoter were developed (Fig 1B). These lines were screened and verified by genotyping using 35S promoter, 3’UTR of almond *PpHKT1*, almond *PpHKT1* specific and *AtHKT1* specific primers (Fig 1). Two constructs of the *PpHKT1* gene, one for overexpression (under the expression of 2x 35S promoter) and one for native expression (under expression of its own native promoter) were transformed into the *athkt1* mutant plants. The T_3_ progenies of the transgenic lines were screened by genotyping and qRT-PCR (Fig 1). Wildtype Arabidopsis plant had amplification of 1.2 Kb fragment of the *AtHKT1* gene with the GABI_795G10_LP/GABI_795G10_RP set of primers specific to the *AtHKT1* gene (Fig 1C). In *athkt1* mutant, *AtHKT1* was interrupted by T-DNA insertion, hence LP/RP primer set was not able to amplify the corresponding fragment (Fig 1C). Genotyping of the transgenic lines expressing *PpHKT1* under native promoter was done by amplification with 3’UTR specific PpHKT1-Start_F and PpHKT1-3’UTR_R primers. Genotyping results showed that the expected amplicon of 1.8 Kb is present in transgenic lines expressing *PpHKT1* under native promoter (PpHKT1NP6) but was absent in transgenic lines overexpressing *PpHKT1* under 2X 35S promoter (PpHKT1OE2.2), WT and the *athkt1* mutant (Fig 1D). Transgenic line overexpressing *PpHKT1* was genotyped by amplification with 2X 35S promoter specific PpHKT1_35S_F and PpHKT1_STOP_R primers. Transgenic lines carrying 2X 35S promoter (PpHKT1OE2.2) amplified 1.7 Kb band in this case, whereas the other lines were missing this fragment band (Fig 1E).

The expression of almond *PpHKT1* was confirmed by qRT-PCR in transgenic lines compared to the mutant *athkt1* plants. Both lines expressing *PpHKT1* in Arabidopsis exhibited statistically significant (P ≤ 0.01) increases in expression compared to the mutant plants (Fig 1F). The overexpression line (PpHKT1OE2.2) displayed 15-fold average increase in the expression compared to the *athkt1* plants (Fig 1F), while the PpHKT1NP6 had a 2.5-fold average increase in the expression compared to the *athkt1* mutant (Fig 1F).

### Salt response of transgenic plants

**Fig 2:**
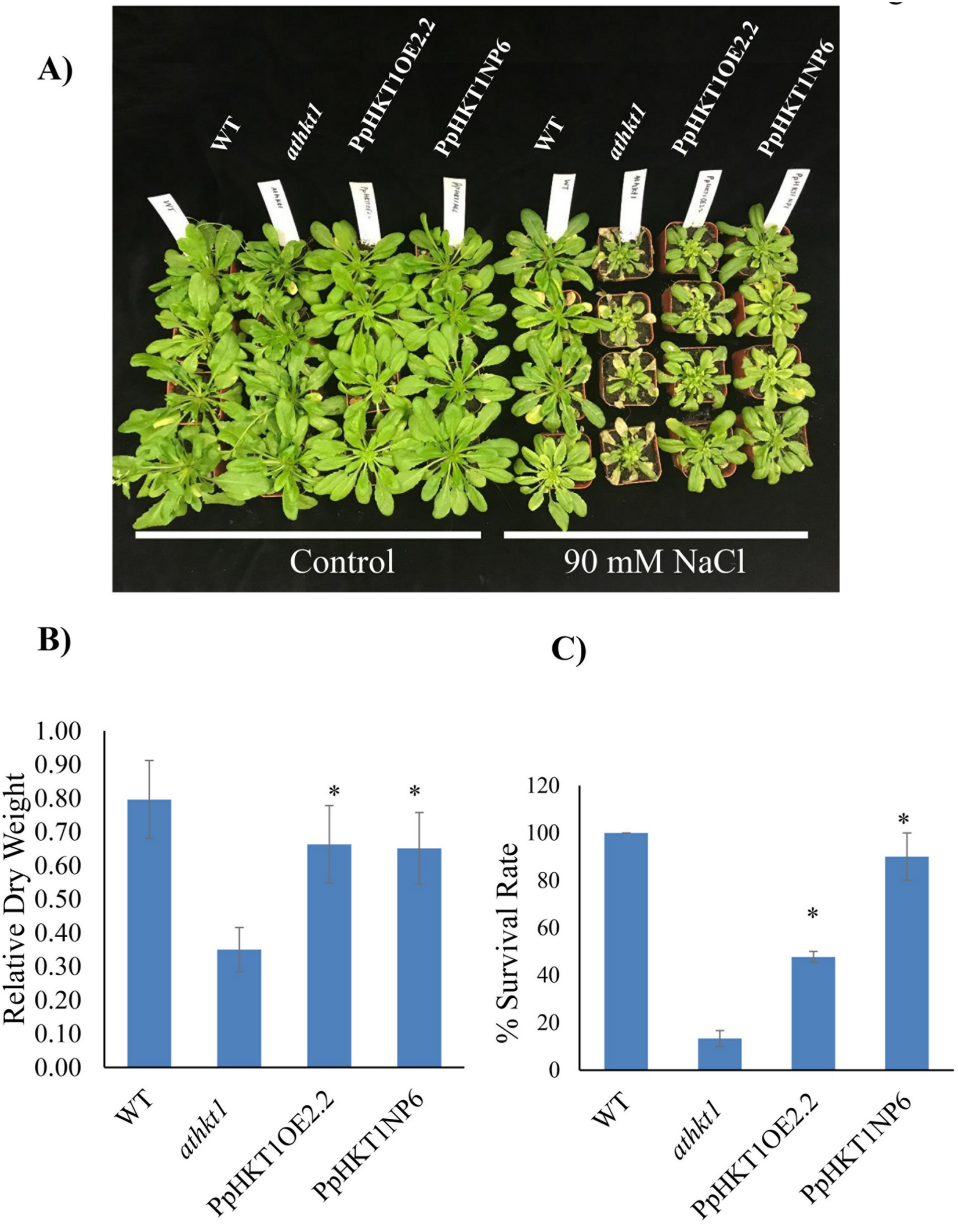
Salt tolerance assays. **A**) WT plants, the *athkt1* mutant and the transgenic lines PpHKT1OE2.2 and PpHKT1NP6 were treated with 90 mM in 1/8-strength-MS media for 18 days. **B**) Plant relative dry weight (DW) of the *athkt1* mutant plants under salinity significantly decreased compared to PpHKT1OE2.2 and PpHKT1NP6 plants. **C**) The transgenic plants showed higher % survival compared to the Arabidopsis mutant *athkt1* after three weeks of salt treatment of 90 mM NaCl. ANOVA single factor was used to depict the statistical differences between the *athkt1* mutant and each of the transgenic lines. P≤0.05 (*).

Several Arabidopsis lines with endogenous- and over-expression of *PpHKT1* were evaluated for salt tolerance by treating them with three salt concentrations (control, 90 mM NaCl and 120 mM NaCl). Mutant *athkt1* plants died with 120 mM NaCl after 18 days of treatment, while PpHKT1OE2.2, PpHKT1NP6 plants and WT plants survived (Data not shown). The transgenic line, PpHKT1NP6, remained greener than PpHKT1OE2.2 and was healthier than the *athkt1* mutant line (Fig 2A). Salinity conferred by 120 mM NaCl was very harsh on Arabidopsis plants resulting in stunted and weak WT, PpHKT1OE2.2 and PpHKT1NP6 plants, thus, for further experiments we selected 90 mM NaCl concentration for salt treatments.

Relative change in dry weight of plants after 18 days of treatment compared with control plants revealed that the *athkt1* mutant had decreased in dry weight under salinity, which was significantly less than the relative change in dry weight of PpHKT1OE2.2 and PpHKT1NP6 (Fig 2B). The transgenic Arabidopsis plants had significantly higher survival rate than the mutant *athkt1* plants. The survival of PpHKT1NP6 plants was similar to WT plants.

**Fig 3:**
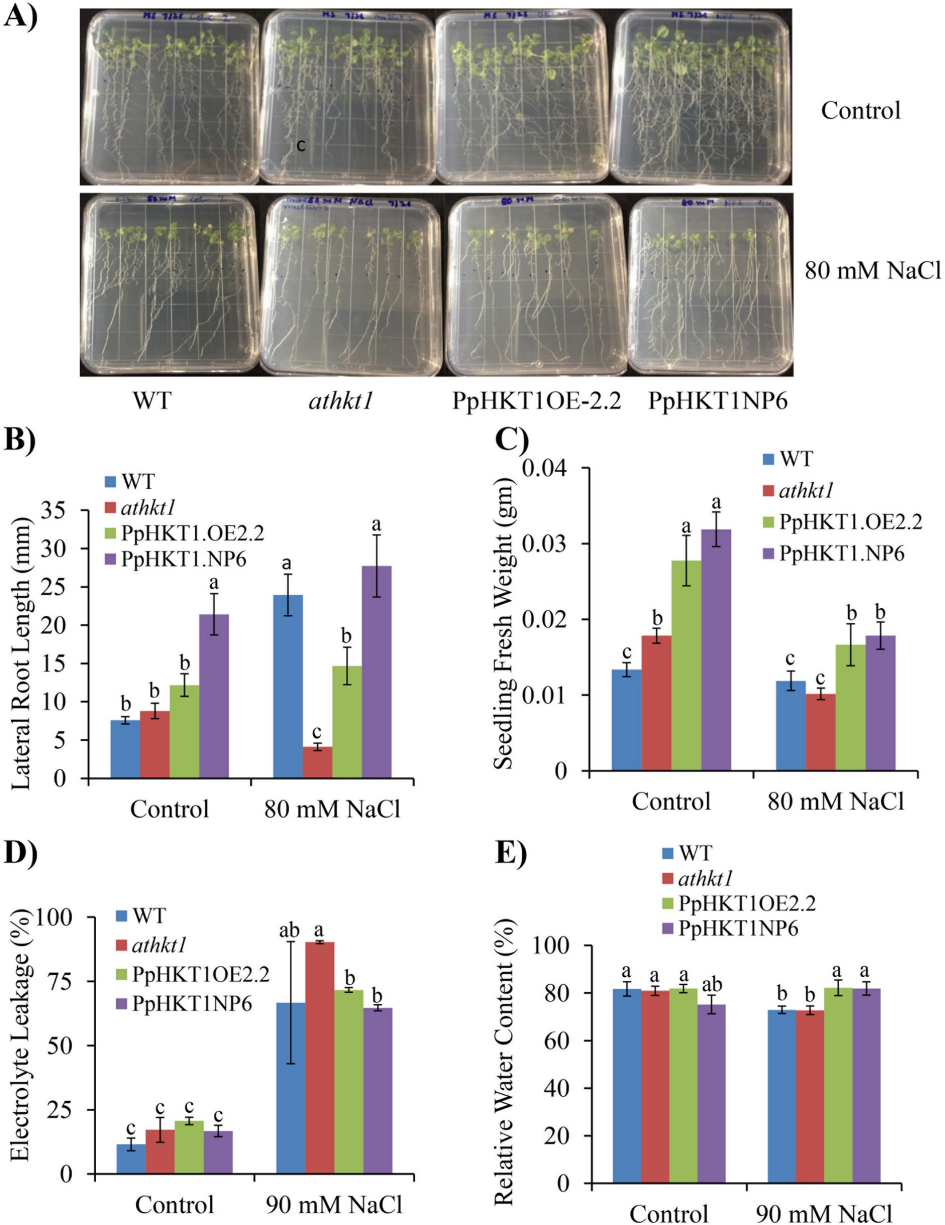
Different salinity stress tolerance parameters evaluated in WT, *athkt1* and the transgenic lines. **A)** Lateral root growth assay. Arabidopsis lines were exposed to 80 mM NaCl and pictured 10 days after treatment. **B)** Average lateral root length calculated by Image J software. **C)** Seedling average fresh weight after 10 days under control or 80 mM NaCl, with 10 seedlings per replicate (n = 3). **D)** Electrolyte leakage calculated 15 days after 90 mM NaCl treatment. **E)** Relative water content calculated 7 days after 90 mM NaCl treatment. ANOVA single factor was used to depict the statistical differences between *athkt1* mutant and each of the transgenic lines. Genotypic means followed by the same letters are not significantly different according to LSD (0.05).

Lateral root growth is a good indicator of salt tolerance in plants. Analysis of transgenic plants for lateral root growth revealed that mutant *athkt1* plants were deficient in lateral root development (Fig 3A). The root length and density were significantly higher in transgenic plants than in mutant plants when 6-day-old seedlings were transferred to 80 mM NaCl in MS media and monitored for 10 days (Fig 3A and 3B). The PpHKT1NP6 showed significant increase in lateral root length as compare to WT, *athkt1* and PpHKT1OE2.2 seedlings under control conditions (Fig 3B). Both PpHKT1NP6 and PpHKT1OE2.2 plants had denser and longer lateral roots compared to *athkt1*, in 80 mM NaCl treatment (Fig 3A and 3B). Of the two transgenic lines, PpHKT1NP6 had denser (Fig 3A) and longer root system (Fig 3B) as compared to PpHKT1OE2.2. The *athkt1* mutant showed a sharp decrease in lateral root growth upon salt stress. The endogenous expression (PpHKT1NP6) stimulated more lateral root growth than the overexpressor (PpHKT1OE2.2) in the control as well as salt treatments (Fig 3B). We also evaluated the FW of seedlings after 10 days of treatment. Although there was a decrease in FW in all lines under salt treatment compared to control, transgenic plants expressing *PpHKT1* had significant increases in seedling FW compared to the *athkt1* mutant (Fig 3C).

Electrolyte leakage is also considered as an indicator of stress and used as a measure to test plants stress response in plants. All four lines showed significantly higher electrolyte leakage in salt treatment compared to the control (Fig 3D). However, in salt treatment, PpHKT1OE2.2 and PpHKT1NP6 had significantly (P ≤ 0.05) lower leakage when compared to *athkt1* (Fig 3D).

Relative water content (RWC) is the measure of water content of a leaf relative to the maximum water it can hold at turgidity and is an indicator of stress. Our analysis showed that both WT and the *athkt1* mutant plants had significantly lower RWC under 90 mM NaCl compared to control plants (Fig 3E). Both transgenic lines PpHKT1NP6 and PpHKT1OE2.2 displayed higher water content then the *athkt1* plants at 90 mM NaCl (Fig 3E). This analysis may indicate that the transgenic plants are under less stress when compared to both WT and mutant plants.

### Salt stress induced expression of *PpHKT1* in Arabidopsis transgenic lines

**Fig 4:**
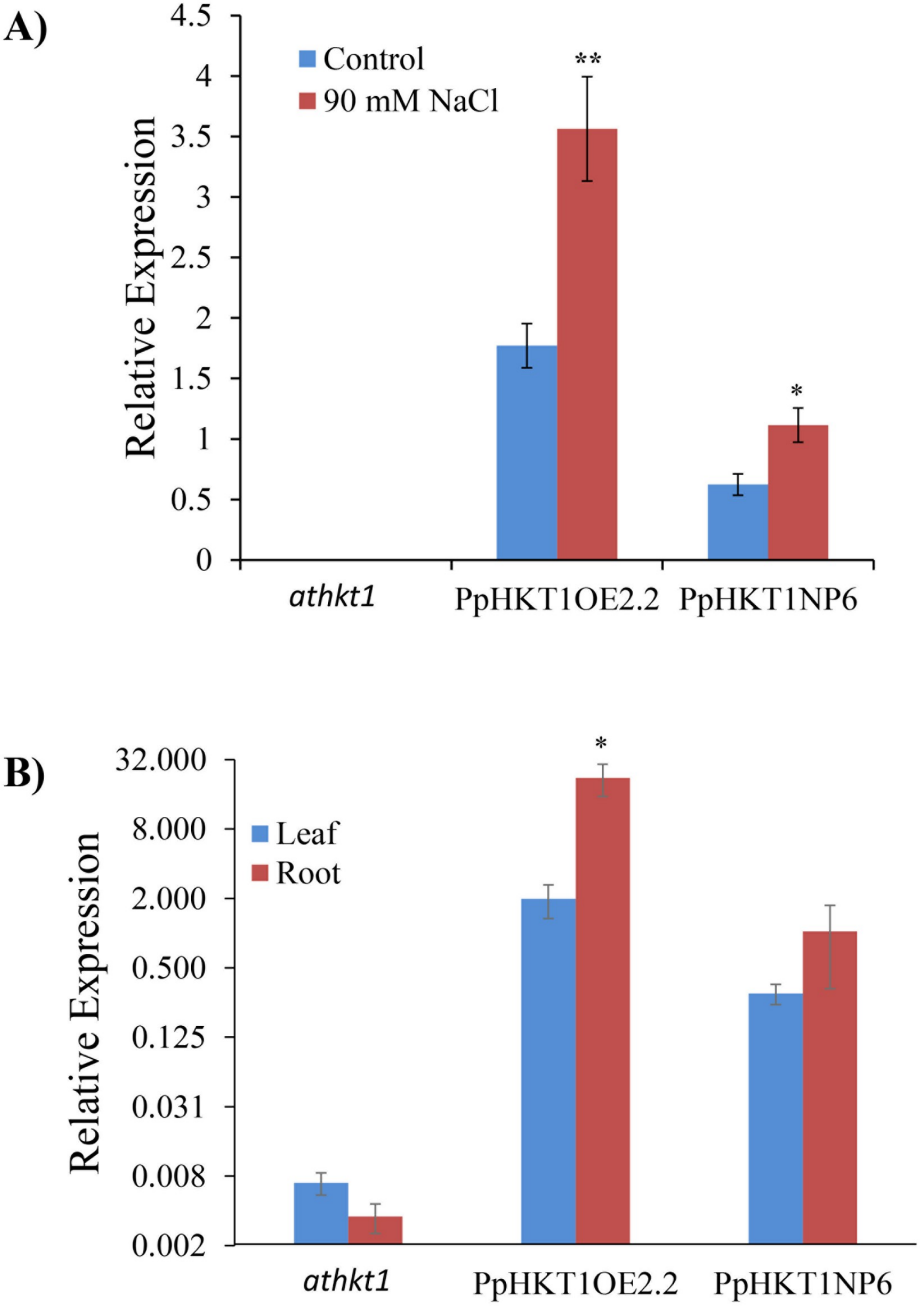
Relative expression of the *PpHKT1* gene in *athkt1*, PpHKT1OE2.2 and PpHKT1NP6. **A**) Under control conditions, the *athkt1* mutant showed no expression while the almond *PpHKT1* variant showed some expression, which was induced under 90 mM NaCl treatment. Samples were collected 48 h after treatment with two seedlings per replicate and three replicates per lines. Experiment was repeated twice. **B**) *PpHKT1* expression in leaves and roots of *athkt1*, PpHKT1OE2.2 and PpHKT1NP6. Expression was significantly more in the PpHKT1OE2.2 roots compared to leaves. ANOVA single factor was used to depict the statistical differences between control and 90 mM NaCl and between leaves and roots. P≤0.05 (*) and P≤0.001 (**).

Both Arabidopsis transgenic lines and the *athkt1* mutant were studied for expression of *PpHKT1* under control and 90 mM NaCl (Fig 4A). *PpHKT1* was not expressed in the Arabidopsis *athkt1* mutant either under control or 90 mM NaCl, while Arabidopsis transgenic lines, expressing the *PpHKT1* gene (*Pp*HKT1OE2.2 and PpHKT1NP6) had significant increases in the expression under treatment (Fig 4A). The *PpHKT1* expressed in leaf and root both in transgenic over expresser and native promoter induced lines, but expression in roots was significantly higher in overexpressor lines as compare to leaves (Fig 4B).

## Discussion

Salinity is the one of the major environmental factors affecting growth and development of plants. The plant deploys various molecular mechanisms to defend itself from excessive salt accumulation in its tissues. Inhibition of the movement of Na^+^ from roots to shoot is a well-known mechanism that is regulated by HKT1 [27]. In this study, we evaluated the role of the *HKT1* gene from almond rootstock ‘Nemaguard’ (*P*. *persica*) during salt stress. ‘Nemaguard’ roots showed induction of *PpHKT1* expression under salt treatment (S1 Fig). For functional validation of *P*. *persica HKT1 (PpHKT1)*, we transformed Arabidopsis *athkt1* mutant GABI_795G10 with the *PpHKT1* gene under its native promoter and a constitutive promoter. The transgenic lines had significantly higher tolerance than the *athkt1* plants under salt treatment (Fig 2). The *athkt1* mutant displayed significant (P ≤ 0.05) decrease in relative plant DW under 90 mM NaCl treatment compared to WT and the transgenic lines PpHKT1OE2.2 and PpHKT1NP6 (Fig 2B). Additionally, the transgenic plants had significantly higher survival rate compared to the Arabidopsis mutant *athkt1* after salt stress (Fig 2C). The PpHKT1NP6 plants, where *PpHKT1* was expressed under native promoter showed almost 90% average survival rate after three weeks of treatment (Fig 2C). As the *athkt1* mutant lacks the ability to restrict the movement of Na^+^ from roots to shoot [7], the enhanced performances of the transgenic lines suggest that PpHKT1 is functionally conserved and helps in reducing the amount of Na^+^ present in the leaves. In rice, *OsHKT1;1*, expressed mainly in the phloem, was unregulated in response to salt stress and was shown to reduce Na^+^ accumulation in leaves by moving Na^+^ to phloem and circulating it back to the roots [28]. In wheat, *TaHKT1;4* was reported to remove Na^+^ from xylem of the leaf sheath and to reduce the amount of Na^+^ accumulation in leaf blades [29]. Later, by comparing salt tolerant and salt sensitive rice cultivars, OsHKT1;4 was shown to restrict movement of Na^+^ from leaf sheath to leaf blade during salt stress [15]. The PpHKT1 protein sequence was used to predict subcellular localization using AtSubP web server (http://bioinfo3.noble.org/AtSubP/?dowhat=AtSubP). The analysis indicates that PpHKT1 contains 9 transmembrane domains and is localized to plasma membrane (S1 Table), which is consistent with its role in regulating Na^+^ concentrations in different plant tissues.

In our investigation, the Arabidopsis transgenic line expressing *PpHKT1* under the native promoter (PpHKT1NP6) exhibited higher survival rate and longer lateral roots under salinity than the overexpressing line PpHKT1OE2.2 (Figs 2C and 3B). It has been shown earlier that the heterologous site-specific expression of *AtHKT1*.*1* in rice root cortex, rather than overexpression, increased plant FW under salinity and reduced accumulation of Na^+^ ions in shoots [30]. The expression of cell type-specific Arabidopsis *HKT1*.*1*, specifically at the mature root stele, reduced the accumulation of Na^+^ in shoot and imparted higher salt tolerance in transgenic Arabidopsis than the overexpressing line [31]. It appears that the regulated site-specific expression of *HKT1* is more effective in coping with salinity stress than its overexpression. The mechanism of HKT1 regulation is unknown so far, and no regulator or protein interactor for HKT1 has been reported yet. Role of post translational modification was reported in Rice HKT1 under salt stress [32]. Recently, it has been shown in *Medicago truncatula* that overexpression of *MtCML40*, a calmodulin-like protein, induced Na^+^ accumulation in shoot and downregulated the expression of MtHKT transporters [33]. So, our results suggest that the significant involvement of other interactors or regulators in the expression of *HKT1* other than promoter mediated induction.

Plants have an extensive root system architecture (RSA) that plays significant role in the adaptation to different stress stimuli. The main component of this root system are lateral roots [34]. The root architecture plays an important role during salt stress [34]. Under salt stress, endogenous abscisic acid (ABA) was shown to promote quiescence in lateral roots as a response to salinity stress [35]. Salt stress is a major inhibitor of lateral root growth, which raises the question of whether salt-tolerance genes such as *HKT1* can help alleviate the salinity stress by stimulating healthy lateral root growth. The transgenic lines expressing *PpHKT1* showed significant increase in lateral root growth under 80 mM NaCl treatment compared to the *athkt1* mutant (Fig 3A and 3B). The role of *HKT1* in lateral root development was also reported earlier where, in a genome-wide association study (GWAS) in Arabidopsis one of the candidate genes involved in lateral root growth under salinity stress was identified as *HKT1* [36]. Lateral root development is a complex trait in plants and is regulated through several different mechanisms. For instance, several miRNA-based regulation mechanisms have been described in several different plants [37, 38]. The *miRNA390* overexpression in poplar (*Populus sp*.) induced lateral root growth and enhanced salt tolerance, whereas *miRNA390* knockout inhibited lateral root growth and compromised salt tolerance [39]. Similarly, soybean plants that had an overexpression of the salt-responsive *miR172c* gene had increased lateral root growth under saline conditions compared to wildtype plants [40]. In Arabidopsis, *miRNA390* regulates the lateral root growth by regulating auxin [37, 38].

Plants try to protect the plasma membrane integrity and permeability during stress [25]. Electrolyte leakage is considered as an indicator of stress and is commonly used as a measure of plant stress tolerance [41]. The electrolyte leakage can be detected instantly after the application of the target stress factor and can last for several hours. The transgenic lines PpHKT1OE2.2 and PpHKT1NP6 exhibited significantly less electrolyte leakage under 90 mM NaCl treatment for 15 days compared to the *athkt1* mutant line. An increase in electrolyte leakage was reported during salinity stress in several plant species including okra (*Abelmoschus esculentus*), cucumber (*Cucumis sativus*), peppers (*Capsicum annuum* L) cultivars, and *Coleus* species [42–45]. Initially, it was believed that this happens due to plasma membrane damage [46], but later it was found that freezing increases membrane permeability to K^+^ and counter ions but does not affect membrane integrity [47]. K^+^ efflux is also shown to be critical during heat and drought stresses [41, 48]. Similarly, several studies reported that K^+^ efflux is a common plant response during salt stress [49]. This study is the first step to understand the role of PpHKT1 in salt stress and its tolerance mechanism. The functional validation of the *PpHKT1* gene in Arabidopsis mutant helped in confirming conservation of HKT1 function in *P*. *persica* and Arabidopsis. Future studies focusing on understanding the roles of other players involved in salt stress in the genus *Prunus* will be vital to improve its salt tolerance. With an increasing scarcity of water due to drought, salinity is becoming a greater problem for almond growers in California. Thus the fate of an almond orchard depends upon the selection of the most robust rootstock, which must be efficient in nutrient uptake and tolerant to various biotic and abiotic stresses. Identification of the key players involved in salt stress and their roles in the mechanism of salt tolerance will help to develop an almond rootstock more suitable for cultivation in salinity-affected regions.

## Supporting information

S1 Fig *PpHKT1* expression in ‘Nemaguard’ roots after 24 h of salt stress. (TIF)

S1 Table Subcellular localization prediction for the PpHKT1 protein using AtSubP web server (http://bioinfo3.noble.org/AtSubP/?dowhat=AtSubP). (DOCX)

## Abbreviations

**cDNA**: **complementary DNA**
CDS: coding DNA sequence
PCR: polymerase chain reaction
qRT-PCR: quantitative Reverse Transcription-Polymerase Chain Reaction
