# Supplementary material for: Expression of the high-affinity K+ transporter 1 (PpHKT1) gene from almond rootstock ‘Nemaguard’ improved salt tolerance of transgenic Arabidopsis
Source: PLoS One. 2019 Mar 26;14(3):e0214473. doi: 10.1371/journal.pone.0214473 (PMC6435114; doi:10.1371/journal.pone.0214473)
Supplement: S1 Table — (DOCX) [file pone.0214473.s002.docx]

| S1 Table. Subcellular localization prediction for the PpHKT1 protein using AtSubP web server (http://bioinfo3.noble.org/AtSubP/?dowhat=AtSubP). | | | | | | | | | |
| --- | --- | --- | --- | --- | --- | --- | --- | --- | --- |
| **Sequence ID** | **Seq length** | **Chloroplast** | **Cytoplasm** | **Golgi** | **Mitochondrion** | **Extracellular** | **Nucleus** | **Cell membrane** | **PREDICTION** |
| **PRUPE.1G067100.1** | 542 | -1.021 | -1.983 | -0.532 | -0.449 | -1.225 | -1.877 | **0.482** | **Cell membrane** |
